# Workplace sexual harassment toward male nurses in South Korea: a cross-sectional online survey

**DOI:** 10.1186/s12912-022-01091-x

**Published:** 2022-11-08

**Authors:** Suyong Jeong, Hyoung Eun Chang

**Affiliations:** 1 Department of Nursing, College of Health and Welfare, Gangneung-Wonju National University, Wonju-si, Gangwon-do South Korea; 2 Research Institute of Nursing Science, College of Nursing, Jeonbuk National University, Jeonju-si, Jeollabuk-do South Korea; 3 Jeonbuk National University, 712 College of Nursing, 567 Baekje-daero, Deokjin-gu, Jeonju-si, Jeollabuk-do 54896 South Korea

**Keywords:** Sexual harassment, Male nurses, Occupational health, Workplace violence, Work environment

## Abstract

**Background:**

Many studies have focused on sexual harassment of female nurses perpetrated by patients and coworkers. However, as males in a female-dominated occupation, male nurses are also at risk of being exposed to sexual harassment. The aim of this study was to examine the prevalence of workplace sexual harassment faced by male nurses in South Korea and to identify related factors.

**Methods:**

A cross-sectional study was conducted using an online survey that recruited male nurses working in various general hospitals in South Korea. In total, 246 participants with at least 6 months of clinical experience enrolled in an online survey, and 155 male nurses were included in the final analysis. Data were collected from May 31, 2019 to July 26, 2020. Items on the questionnaire included sexual harassment experiences, nursing work environment, and general characteristics of the participants. Descriptive statistics were used to analyze participants’ general characteristics and a negative binomial regression model was used to analyze predictors of workplace sexual harassment.

**Results:**

The mean number of sexual harassment incidents was 3.2 ± 5.5. The majority (65.2%) of male nurses had experienced sexual harassment at least once at work. The negative binomial regression model in the study was found to be acceptable (likelihood ratio chi-square = 30.03, *df* = 18*, p* = .037). The perceived nursing work environment was the only significant predictor of sexual harassment towards male nurses (*p* = .001; incidence rate ratio = 0.37; 95% CI, 0.20–0.66).

**Conclusions:**

Nursing administrators must recognize that men in female-dominated occupation may experience sexual harassment in the workplace. Specific and realistic managerial policies and educational programs should be implemented to prevent workplace sexual harassment and improve the nursing work environment for male nurses.

## Background

Sexual harassment (hereafter SH) in the workplace refers to unwelcomed physical, verbal, or non-verbal conduct in the workplace that makes the victim feel humiliated or uncomfortable about sexual matters [1]. Harassment has been classified according to its severity as sexual coercion, unwanted sexual behavior, and gender harassment [2]. Regardless of gender, workplace SH is a social problem that disturbs a healthy working environment and lowers the overall quality of life for workers [3]. Measuring and monitoring SH are challenging tasks because of the pervasiveness of harassment and underreporting by victims [1]. Therefore, it is important to make efforts at the organizational level to enhance gender equality that are supported by empirical evidence regarding the actual occurrence of workplace SH. The reported causes of SH are vertical gender discrimination and power imbalance, isolation and private relationships involving intimate interactions, and economic instability [4].

According to a study that systematically reviewed 5320 SH studies from 1977 to 2020, workplace SH has been widely addressed by studies in a variety of fields such as the military, school, and healthcare [5]. Although the frequency and characteristics of SH varies widely, depending on differences in culture, education level [6], and nationality [1], the occurrence of SH tends to be higher in more gender-typed occupations, in which one gender predominantly outnumbers the other [7]. Numerous studies have investigated workplace SH over the decades; however, less attention has been paid to male victims than to female victims, even though the damage caused by SH is detrimental regardless of the victim’s gender [7]. The nursing sector may be more susceptible to SH than other workplaces due to isolated work settings and the intimate nature of patient care [8]. Nursing jobs traditionally have a high proportion of women to men, with a vertical relationship between female supervisors and male subordinates. Therefore, male nurses are vulnerable to SH. The male nursing workforce requires action at the organizational level to prevent SH.

Many studies have focused on male nurses in terms of general gender-related issues, such as the difficulties of adjusting to the female-dominated organizations, rather than SH victimization [9–14]. These qualitative studies investigated topics such as the social context of nursing as a man among women [9, 10], the gender issues experienced by male nurses in the workplace [9–14], and acceptance by female colleagues and patients [12]. In a qualitative study on SH among Korean male nurses, participants described experiences of SH that were too subtle for them to recognize their victimization themselves [13].

Although quantitative evidence on SH in nursing is abundant [14], related research has focused predominantly on only female nurses [6, 15] or both female and male nurses [16–18]; in contrast, there remains a lack of research on SH specifically among male nurses [19]. These quantitative studies have used convenience sampling rather than representative (random) sampling because SH is a sensitive issue [19]. The main topics were the prevalence, causes, and outcomes of workplace SH. Prevalence estimates of SH were well-documented in a recent meta-analysis of research on SH against nurses worldwide [14]. According to the findings, the incidence of SH toward nurses over a 12-month period was 12.6% and the incidence of SH over the span of a clinical nursing career was 53.4%, which was higher than the incidence of SH in the general workplace. Those findings indicated that a higher proportion of male nurses than female nurses reported SH.

Even though SH data are count data, which have a skewed or overdispersed distribution, most researchers have treated SH as a binary variable and primarily analyzed the frequency and causes of SH using logistic regression models [3]. This application not only leads to a loss of valuable information, but also reduces the model fit [20]. To solve these issues, negative binomial regression models have been identified as better than other analytic methods when dealing with low-frequency count data, such as in studies of SH or sexual aggression [21].

It is important to consider organizational environments as antecedent situational factors that affect SH. According to the theoretical framework of SH, organizational factors include organizational atmosphere, gender ratio, and gender in the context of the job [22]. Finally, SH negatively affects both physical and mental health outcomes, as well as job-related outcomes such as productivity, job satisfaction, organizational commitment, and turnover [23, 24]. Many recent studies have found that the quality of the nursing work environment was a critical influence on improving nurses’ outcomes (job satisfaction and organizational commitment) and quality of care [25, 26]. Concomitantly with these research trends, measurement of the nursing work environment has emerged as a significant issue, and the Practice Environment Scale of Nursing Work Index (PES-NWI) was developed by monitoring measurable indicators in magnet hospitals [27]. The PES-NWI has been used in various settings with modifications to fit the clinical and organizational contexts of each country [28].

In summary, there is a lack of quantitative evidence for the prevalence of SH, causes of SH, and coping mechanisms of male nurse SH victims. Although quantitative studies on workplace SH in the nursing field are abundant, those studies have focused on predominantly female nurses and had limitations in terms of the analytic technique because they treated SH data as a binary variable. Therefore, it was necessary to accurately and quantitatively explore factors that could decrease SH and provide a snapshot of male nurses’ circumstances in order to prepare evidence-based measures for nursing managers. This study aimed to analyze the SH experienced by male nurses in South Korea by investigating the prevalence, coping responses, and causes of workplace SH and to offer practical suggestions and strategies for nursing management.

## Methods

### Design and procedure

This study was designed as a correlational descriptive study, using a cross-sectional online survey to explore the factors that influence the SH experience of male nurses. The subjects of this study were male nurses who had worked for at least 6 months in general hospitals. Convenience sampling was conducted of those who voluntarily agreed to complete the questionnaire. An online survey link was provided to the subjects through recruitment posts distributed through Korean hospitals, or on social media channels that many Koreans use and access. To ensure the representativeness of the study sample, snowballing recruitment procedures were applied. We were eventually able to collect final samples that were fairly geographically balanced across the country, and between metropolitan and non-metropolitan areas (metropolitan areas included Seoul, Incheon, and Gyeonggi Province, whereas non-metropolitan areas included Busan, Daejeon, Gangwon Province, South Chungcheong Province, North Gyeongsang Province, South Gyeongsang Province, and Jeju Province). This study received ethical approval from the Institutional Review Board of the institution where the author was affiliated (IRB no.: 2019–228-01). Data were collected from May 31, 2019, to July 26, 2020. The online questionnaire included written consent for providing and utilizing personal information. The questionnaire was distributed to a total of 246 people, and the final sample used for analysis was 155 people. Ninety-one people were excluded for the following reasons: 39 due to disagreement with participation, 17 because there was no response regarding SH experiences, 30 due to missing values, 4 because of duplications, and 1 because the respondent was not qualified (not currently working as a nurse).

### Variables and instruments

#### Dependent variable: sexual harassment

The Sexual Harassment Questionnaire–Department of Defense short version (SEQ-DoD-s), developed by Stark and colleagues [29], was used to measure experiences of SH in this study. The authors obtained permission from the developer and performed translation from English to Korean and back-translation. This tool included four subfactors with a total of 16 items: sexist behavior (4 items), crude/offensive behavior (4 items), unwanted sexual attention (4 items), and sexual coercion (4 items). In the study by Stark et al. [29], Cronbach’s alpha was .92, while in this study, Cronbach’s alpha was .92, ranging from .78 to .87 in all subfactors. Each item of the SEQ-DoD-s was measured on a 5-point response scale (coded as 0 = never, 1 = once or twice, 2 = sometimes, 3 = often, 4 = many times). The instrument comprised questions on SH exposure during the past 6 months. The total SEQ-DoD-s score and frequency of SH were calculated by totaling the scores of each polytomous item. High scores indicated high levels of exposure to SH. The survey questions on the SEQ items used in the present study can be found in Table 2. Additional questions were included to investigate the coping responses of those who were sexually harassed. The additional items included information on the perpetrator, coping responses, whether the incident was reported, and the reasons for not reporting the incident.

#### Independent variable: perceived nursing work environment

To measure the perceived nursing work environment, we used the Korean translation of the Practice Environment Scale of the Nursing Work Index (PES-NWI) questionnaire [30], which Lake [27] originally developed. With a total of 29 items, this tool consists of 5 subfactors: nurse participation in hospital affairs (9 items); nursing foundations for quality of care (9 items); nurse managers’ ability, leadership, and support of nurses (4 items), adequacy of staffing and resources (4 items); and collegial nurse-physician relations (3 items). Cronbach’s alpha from Cho et al. [30] was .93, and Cronbach’s alpha for this study was .92, ranging from .80 to .84 in all subfactors. Each item of the PES-NWI was measured on a 4-point Likert scale (coded as 1 = strongly disagree, 2 = disagree, 3 = agree, 4 = strongly agree). High scores corresponded to high positive perceptions of the nursing work environment.

#### Independent variable: general characteristics

Data on general characteristics were divided into the individual, hospital, and unit levels. Individual-level variables included age (coded as 1 = 20–29 years, 2 = 30–39 years, 3 = 40+ years), marital status (coded as 1 = married, 2 = unmarried), and education level (coded as 1 = associate degree, 2 = bachelor’s degree, 3 = master’s or higher), and years of clinical experience (coded as 1 = < 1 year, 2 = 1- < 3 years, 3 = 3- < 5 years, 4 = 5+ years). Hospital-level variables included location (coded as 1 = metropolitan area, 2 = non-metropolitan area) and number of hospital beds (coded as 1 = < 500, 2 = 500–999, 3 = 1000+). Unit level variables included job position (coded as 1 = charge nurse or head nurse, 2 = staff nurse), type of unit (coded as 1 = medical or surgical ward, 2 = special unit such as the emergency room, intensive care unit, operating room, outpatient department, administration department, physician’s assistant or others), years of current unit experience (coded as 1 = < 1 year, 2 = 1- < 3 years, 3 = 3- < 5 years, 4 = 5+ years), shift pattern (coded as 1 = non-shift work, 2 = shift work), and perceived nursing work environment (continuous).

### Data analysis

Descriptive statistics were utilized for general characteristics and SH experiences. The significance level for all hypothesis tests was a *p-*value < 0.05. Since the data of the total SEQ-DoD-s score had a positively skewed distribution and overdispersion, data were treated as count variables. To analyze factors associated with SH, we performed a non-negative binomial regression analysis including all independent variables.

## Results

### General characteristics of the study sample

In total, 155 male nurses were included in the final analysis (Table 1). The mean age was 31.0 years, 76.1% were unmarried, and 87.2% had a bachelor’s degree. More than half reported that their hospitals were in metropolitan areas and had over 1000 beds. Nursing roles included 82.1% practicing staff nurses, compared with 10.9% in managerial positions such as charge nurse or head nurse. Male nurses who worked in general medical or surgical wards were much fewer than those in special units, such as the emergency room, intensive care unit, and operating room. Participants had an average of 4.5 ± 4.1 years of total clinical experience and 2.9 ± 2.6 years’ experience in their current unit. The subjects who worked shifts accounted for 69.7% of the total sample. The mean score of the perceived nursing work environment was 2.64 ± 0.44 on a 1–4 scale.

**Table 1:** General characteristics of male nurses in a study of workplace sexual harassment (*N* = 155)

Variables	Categories	Item	n (%)	Mean ± SD
Individual level	Age (years)	20–29	74(47.7)	31.0 ± 4.8
		30–39	69(44.5)	
		40+	12(7.7)	
	Marital status	Married	37(23.9)	
		Unmarried	118(76.1)	
	Educational level	Associate degree	5(3.2)	
		Bachelor’s degree	136(87.7)	
		Master’s or higher	14(9.0)	
	Years of clinical experience	< 1	18(11.6)	4.5 ± 4.1
		1- < 3	53(34.2)	
		3- < 5	38(24.5)	
		5+	46(29.7)	
Hospital level	Location	Metropolitan area	108(69.7)	
		Non-metropolitan area	47(30.3)	
	Beds	< 500	16(10.3)	
		500–999	54(34.8)	
		1000+	85(54.8)	
Unit level	Job position	Charge nurse or head nurse (Managerial position)	19(12.3)	
		Staff nurse	136(87.7)	
	Unit	Ward (medical/surgical)	47(30.3)	
		Special (ER, ICU, OR, OPD, administration, PA)	108(69.7)	
	Years of current unit experience	< 1	39(25.2)	2.9 ± 2.6
		1- < 3	58(37.4)	
		3- < 5	36(23.2)	
		5+	22(14.2)	
	Shift pattern	Non-shift	47(30.3)	
		Shift	108(69.7)	
	Perceived nursing work environment (4 points)			2.64 ± 0.44

*ER* Emergency room, *ICU* Intensive care unit, *OR* Operating room, *OPD* Outpatient department, *PA* Physician assistant

### Assessment of workplace sexual harassment

Sexual harassment data were found to be highly right-skewed (skewness = 4.1). The median and interquartile range of the total SEQ-DoD-s scores were 2.0 and 4.0, respectively. The frequency at which male nurses experienced SH was 3.2 ± 5.5 during the past 6 months. From the study sample, 65.2% responded that they were exposed to SH at least once, whereas 34.8% did not experience any SH.

Table 2 shows the results of analyzing the SEQ-DoD-s data as binary or count variables. Among the SEQ-DoD-s items, the highest percentage (57.4%) of participants answered “yes” to the item “Treated you differently because of your gender (for example, mistreated, slighted, or ignored you)?” and the lowest percentage (1.9%) answered “yes” to “Treated you badly for refusing to have sex?”. In general, the frequencies of experiences in the sexual behavior (1.6 ± 2.0) and crude/offensive behavior (1.0 ± 1.8) categories were greater than those of experiences in unwanted sexual attention (0.5 ± 1.5) and sexual coercion (0.2 ± 1.2) categories.

**Table 2:** Assessment of workplace sexual harassment (*N* = 155)

Categories of Sexual Harassment		Responses (%)						Frequency
		No	Yes	Once or twice	Sometimes	Often	Many times	
Total		34.8	65.2					3.2 ± 5.5
*Sexist Behavior*								1.6 ± 2.0
1	Referred to people of your gender in insulting or offensive terms?	83.2	16.8	9.7	5.8	1.3	–	
2	Treated you “differently” because of your gender (for example, mistreated, slighted, or ignored you)?	42.6	57.4	21.3	29.7	6.5	–	
3	Made offensive sexist remarks (for example, suggesting that people of your gender are not suited for the kind of work you do)?	92.9	7.1	3.2	3.2	0.6	–	
4	Put you down or was condescending to you because of your gender?	84.5	15.5	9.7	3.9	1.9	–	
*Crude/Offensive Behavior*								1.0 ± 1.8
5	Repeatedly told sexual stories or jokes that were offensive to you?	68.4	31.6	18.7	10.3	2.6	–	
6	Made unwelcome attempts to draw you into a discussion of sexual matters (for example, attempted to discuss or comment on your sex life)?	93.5	6.5	5.2	1.3	–	–	
7	Made offensive remarks about your appearance, body, or sexual activities?	76.1	23.9	16.8	4.5	2.6	–	
8	Made gestures or used body language of a sexual nature that embarrassed or offended you?	93.5	6.5	5.2	–	0.6	0.6	
*Unwanted Sexual Attention*								0.5 ± 1.5
9	Made unwanted attempts to establish a romantic sexual relationship with you despite your efforts to discourage it?	93.5	6.5	1.9	3.9	0.6	–	
10	Continued to ask you for dates, drinks, dinner, etc., even though you said “No?”	93.5	6.5	5.2	0.6	–	0.6	
11	Touched you in a way that made you feel uncomfortable?	87.1	12.9	10.3	1.9	0.6	–	
12	Made unwanted attempts to stroke, fondle, or kiss you?	94.8	5.2	1.9	2.6	0	0.6	
*Sexual Coercion*								0.2 ± 1.2
13	Made you feel like you were being bribed with some sort of reward or special treatment to engage in sexual behavior?	97.4	2.6	0.6	1.3	0.6	–	
14	Made you feel threatened with some sort of retaliation for not being sexually cooperative (for example, by mentioning an upcoming review)?	96.1	3.9	2.6	0.6	0.6	–	
15	Treated you badly for refusing to have sex?	98.1	1.9	1.3	–	0.6	–	
16	Implied faster promotions or better treatment if you were sexually cooperative?	97.4	2.6	1.3	0.6	–	0.6	

### Coping responses when sexually harassed

Coping responses to workplace sexual harassment are presented in Table 3. Among the 101 male nurses who experienced SH at least once, 67 responded to the item about the perpetrator; the most common perpetrators were nursing colleagues (49.3%), followed in descending order by patients (34.3%), nursing managers or supervisors (10.4%), patients’ family members (3.0%), and doctors (1.5%). A total of 96 subjects answered the question “When sexually harassed, how did you cope with it?”, with common responses including “just laughed it off” (32.3%), “did not express anything” (12.5%), and “avoided the situation” (9.4%), although 15.6% expressed their displeasure.

**Table 3:** Coping responses to workplace sexual harassment

Variable	Item	n	%
Perpetrator (*n* = 67)	Nursing colleagues	33	49.3
	Patients	23	34.3
	Nurse managers or supervisors	7	10.4
	Patients’ family members	2	3.0
	Doctor	1	1.5
	Others	1	1.5
Coping responses when harassed (*n* = 96)	Laughed it off	31	32.3
	Expressed their displeasure	15	15.6
	Did not express anything	12	12.5
	Avoided the situation	9	9.4
	Others	29	30.2
Did you report it when harassed? (*n* = 101)	Yes	9	8.9
	No	92	91.1
Reasons why they did not report when harassed (*n* = 56)	I did not think that there would be any change even if reported	33	58.9
	It is tiresome	17	30.4
	I am afraid of retribution	3	5.4
	I do not know where to report	2	3.6
	Hospital did not have any reporting system	1	1.8

Of the 101 male nurses, 91.1% did not report the incidents when sexually harassed. To the question of why they did not report, 56 responded. Answers included “because I did not think that there would be any change even if reported” (58.9%), “because it is tiresome” (30.4%), “because I am afraid of retribution” (5.4%), “because I do not know where to report” (3.6%), and “because hospital did not have any reporting system” (1.8%).

### Factors associated with sexual harassment towards male nurses

The negative binomial regression model in our study (Table 4) was found to be acceptable (likelihood ratio chi-square = 30.03 *df* = 18, *p*-value = .037) since the omnibus test indicated that our model (dispersion parameter = 1.45, deviance value/*df* = 1.20) was superior to the null model (only the intercept). Furthermore, the goodness of fit value for our regression model (log likelihood = − 334.52, Akaike information criterion [AIC] = 709.05, Bayesian information criterion [BIC] = 769.92), was better than that of the Poisson log-linear model (log likelihood = − 529.61, AIC = 1097.22, BIC = 1155.05). According to the coefficients of our model, perceived nursing work environment was the only significant predictor of SH toward male nurses (*p*-value = .001, incidence rate ratio = 0.37, 95% CI 0.20–0.66). Interpreting these findings, the likelihood of having been sexually harassed in the workplace decreased by 63% if the score of the perceived nursing work environment increased by 1 point.

**Table 4:** Factors associated with workplace sexual harassment using negative binomial regression (*N* = 155)

Variables	Parameter	B	SE	Wald Chi-Square	p-value	Incidence rate ratio	95% CI for incidence rate ratio	
							Lower	Upper
**Individual level**	(Intercept)	4.03	1.48	7.46	.006	56.42	3.12	1020.05
Age (ref. 20–29 years)	40+	−.85	0.67	1.61	.204	0.43	0.12	1.59
	30–39	−.87	0.46	3.54	.060	0.42	0.17	1.04
Marital status (ref. married)	Unmarried	−.54	0.34	2.51	.113	0.58	0.30	1.14
Educational level (ref. associate degree)	Master or PhD	1.15	0.85	1.82	.177	3.16	0.60	16.75
	4-year college	.42	0.76	.30	.584	1.52	0.34	6.76
Years of clinical experience (ref. < 1 year)	5+	−.17	0.67	.06	.806	0.85	0.23	3.16
	3- < 5	−.15	0.62	.06	.811	0.86	0.26	2.90
	1- < 3	−.63	0.58	1.19	.276	0.53	0.17	1.66
**Hospital level**								
Location (ref. metropolitan area)	Non-metropolitan area	−.50	0.35	1.97	.160	0.61	0.30	1.22
Beds (ref. < 500)	1000+	−.40	0.49	.66	.415	0.67	0.25	1.76
	500–999	.19	0.48	.16	.692	1.21	0.47	3.12
**Unit level**								
Job position (ref. charge or head nurse)	Staff nurse	−.25	0.47	.28	.599	0.78	0.31	1.98
Unit (ref. medical/surgical ward)	Special units	.38	0.27	1.93	.165	1.46	0.86	2.50
Years of current unit experience (ref. < 1 year)	5+	−.55	0.61	.82	.365	0.58	0.17	1.91
	3- < 5	.67	0.48	1.99	.158	1.96	0.77	4.98
	1- < 3	.38	0.44	.74	.390	1.46	0.62	3.48
Shift pattern (ref. non-shift)	Shift	.49	0.30	2.54	.111	1.62	0.89	2.95
Perceived nursing work environment (4 points)		−1.01	0.30	10.98	.001	0.37	0.20	0.66

## Discussion

This study, to the best of our knowledge, is the first to explore the frequency and causes of SH toward male nurses working in various areas and hospitals across South Korea for the purpose of providing nursing managers and policymakers with empirical evidence. The findings revealed a high prevalence of SH, with 65.2% of male nurses having experienced SH at least once during the past 6 months at their workplace.

The prevalence of SH among male nurses in this study was higher than the previously reported proportions of 40% among Greek male nurses [18, 19] and 34% among Australian male nurses [17]. However, simple comparisons of the prevalence of SH among countries and studies are difficult and can be misleading [15]. Methodological differences including different measurement tools or questionnaires, differing time spans of exposure to SH, variation in social and cultural characteristics, and differences in hospital settings, as well as individual perceptions and understanding of SH, can lead to inconsistent results for levels of SH across studies [14, 15].

Our results showed that the most frequent type of SH was sexual behavior and crude/offensive behavior (these two types are collectively called gender harassment), whereas the least frequent type of SH was sexual coercion. These findings are consistent with those of previous studies conducted on Greek male nurses [18, 19]. Within gender harassment, our study showed that mistreatment because of gender, repeated sexual stories or jokes, and comments about appearance were the most frequent forms of SH. The most frequent type of SH perpetrator was a nursing colleague (49.3%) or a patient (34.3%). Not only could male nurses be sexually harassed by nursing colleagues in a hostile work environment and a female-dominated organization, but they could also be harassed by patients and their family members while deeply involved in care [4]. Nursing managers should monitor the occurrence and types of SH (especially gender harassment) experienced by men and take actions to reduce SH in the workplace. Maghraby et al. [15] suggested that nurses should be educated repeatedly on how to protect themselves, and managers should provide patients and visitors with informative materials concerning SH policies within their organization.

Our findings revealed that more than half of male nurses remained passive in their response to SH, “laughing it off” (32.3%), “not expressing anything” (12.5%), and “avoiding the situation” (9.4%), whereas 15.6% of respondents actively responded and expressed their displeasure. In addition, the majority of male nurses (91.1%) did not report SH incidents, with the most frequent reason for not reporting (58.9%) being that they did not expect reporting to result in any change. These findings were similar to previous studies reporting “male nurses’ silence,” a phrase describing a phenomenon where men prefer not to report SH due to indifference by others toward their situation and concerns about relationships with coworkers [18]. Considering that masculinity can make it difficult for male victims to disclose their sexual victimization because of the shame [31], underreporting of SH among male nurses could be a particularly serious problem, no less than for female nurses. Moreover, male nurses in patriarchal cultures may experience greater suffering due to those gender roles and expectations for stronger masculinity. In summary, although the prevalence of SH towards male nurses may be high, it may not be easy for nursing managers to identify the victims because male nurses are reluctant to make official reports. Nursing managers should establish formal confidential reporting systems and support male nurses in voluntarily reporting any SH incident. In addition, nurses should eliminate discrimination against men in the workplace, reduce prejudice against male nurses, and enhance gender sensitivity.

Identifying the predictors of SH is necessary for establishing effective policies. This study showed that the perceived nursing work environment was the only significant predictor of workplace SH, while all other independent variables were not statistically significant. These results are inconsistent with previous studies showing that age and clinical experience were the main factors influencing SH [14, 15, 18, 32]. This was probably because our study sample had less socio-demographic balance than other studies, given that our participants were concentrated in younger age groups and had less clinical experience.

Our findings showed that when perceived nursing work environment improved, the prevalence of SH in the workplace decreased dramatically. Although causal relationships could be inferred from various theoretical models and empirical studies, we could not compare our study directly to previous studies due to the lack of evidence for causal relationships between the nursing work environment and SH. From a theoretical standpoint [22], the causes of SH are imbalances of power and status (organizational models), mismatched gender-based expectations (sex-role spillover model), and individual predisposition combined with organizational norms (person-environment model). Considering this theoretical background, the perceived nursing environment in this study could perhaps be recognized as a mixture of personal perceptions and organizational norms around nursing work (a person-environment model) among men who worked in the non-traditional and female-dominated sectors (a sex-role spillover model) and had fewer years of experience and younger age (an organizational model). This interpretation can be supported by empirical evidence reporting a negative association between the nursing work environment and nurses’ outcomes such as harassment [33, 34] and workplace bullying [35]. Thus, nursing managers should monitor male nurses’ perceptions of the work environment and improve the nursing work environment by developing nursing supervisors’ managerial abilities and leadership skills, supporting and encouraging male nurses’ participation in hospital affairs and quality of care, and ensuring adequate staffing, resources, and human relations within the organization.

This study had several limitations. First, our study findings do not represent the general Korean male nurse population because convenience sampling was used. Second, causal relationships in our study cannot be determined due to the cross-sectional study design. Despite these limitations, our findings add data about the association between the perceived nursing work environment and SH in male nurses. Future studies need to recruit a larger and more diverse population of male nurses for a longitudinal study to further investigate the causes and effects of SH, the predictors of SH across multiple clinical settings and areas, and the physical, mental, and job consequences of SH.

## Conclusions

This study contributes to an understanding of SH toward male nurses in South Korea and of the interventions needed from nursing managers and policymakers, furthering the empirical evidence about workplace SH towards men in non-traditional and female-dominated fields. It is necessary to prevent SH by effectively managing organizational variables, especially the nursing work environment for male nurses. In conclusion, nursing managers should make efforts to improve the nursing work environment and to prevent SH toward male nurses by monitoring the occurrence of SH, establishing protocols and guidelines that encourage voluntary reporting of sexual misconduct, and prohibiting SH in the workplace.

## Data Availability

The datasets generated during and/or analyzed during the current study are available from the corresponding author on reasonable request.

## Abbreviations

SH: Sexual Harassment
PES-NWI: Practice Environment Scale of Nursing Work Index
SEQ-DoD-s: Sexual Harassment Questionnaire–Department of Defense short version
AIC: Akaike Information Criterion
BIC: Bayesian Information Criterion
ER: Emergency Room
ICU: Intensive Care Unit
OR: Operating Room
OPD: Outpatient Department
PA: Physician Assistant
CI: Confidence Interval
